# Treatment of Pulpless Teeth

**Published:** 1886-04

**Authors:** J. W. Jay


### Treatment of Pulpless Teeth.

BY J. W. JAY.

Read before the Mississippi Valley Dental Society.

There is hardly any topic more common, or one with which the dentist comes in contact, than the one proposed for our present consideration. And yet, for all that, to one who is seeking the very best possible interests of those, who are willing to place themselves in his hands for treatment, and who is desirous of guarding his own reputation, it never ceases to be an interesting one.

The latitude that I propose to range over will be circumscribed by two conditions, first, a pulpless tooth without an alveolar abscess, and secondly, one with an abscess. Now, in order to make the case as convenient as possible; and one in which we can be as readily assured of success, as any we can mention, we will suppose these conditions applicable to one of the central incisors.

The first case is a pulpless tooth without the accompanying abscess, in which all seems to be "quiet on the *Potomac*," so far as any inflammation is concerned.

In a case like this, it is important that two leading facts be considered, to say nothing of others that not unfrequently are apparent, before we enter too far into the domain that we are about to explore; to ascertain as nearly as possible the length of time since the death of the pulp, and the sanitary condition of the patient.

It may have been so long since the death of the pulp occurred, and the investing membrane of the root in such an unhealthy condition, and as a consequence, the disintegration of the bone structure of the tooth so far advanced, as to render all treatment and the hope of saving the tooth utterly abortive, let the sanitary condition of the patient be never so good.

But, if the death of the pulp has been of recent date, and the contiguous parts, so far as we can perceive, in a normal condition, then we may have a reasonable hope of success, in saving the tooth for a series of years. In a case like this, we are going on the ground that there is decay on one of the approximal surfaces that has penetrated to the pulp and serves as a pocket to collect and hold extraneous matter, the chemical decomposition of which, has, in all probability, been the exciting cause of the death of the pulp.

Usually, on examination of a tooth like this, we find the pulp chamber and nerve channel packed with debris, the accumulations of weeks, months, and even years, presenting to the eye a putrescent tank, that if agitated a little, sends out an "ordoriferous effluvia," which, to the olfactories, is comparable to what might be expected to arise from a certain kind of sepulchre alluded to in Holy Writ.

Of course the first thing to be done is to thoroughly cleanse the tooth, and this can be done by carefully opening the canal with a hand drill or burring engine and washing out the cavity with a solution of the chloride of sodium, or a preparation of carbolic acid, glycerine, and rain water.

After the baptism above indicated, the cavity should be wiped as dry as possible with cotton, or some other drying material, and then on the point of a broach introduce a small pledget of cotton saturated with the oil of eucalyptus, creosote, or carbolic acid, withdrawing the pledget, at the same time being sure to leave a sufficient quantity of the fluid to thoroughly moisten the whole surface of the cavity, and especially at the apex of the root. This being satisfactorily accomplished, fill as loosely as possible with dry cotton to prevent the ingress of foreign matter, to remain there and ferment, and increase the very condition you are endeavoring to overcome, and dismiss the patient with the injunction to call in a few days.

It is well enough just here, however, to remember, and he who does not, will sometimes, sooner or later, wake up to a glorious consciousness of his forgetfulness in a manner that will keep his latent memory jogged for the balance of the time of his dental probation; that it often happens on opening up such a tooth, that an inflammatory action of the periosteum, more or less severe, ensues, and sometimes becomes very annoying to the patient, as well as to the operator.

Of a truth, I might say this is almost sure to be the result, if the cavity is tightly closed at the apex or crown surface of the tooth, immediately after being opened up.

It will be necessary to continue the treatment as I have indicated, varying it, of course, with other remedies, that in the lapse of time and experience may be suggested to the mind of the operator, until the sanitary conditon of the tooth and its surroundings are such as to warrant the finishing of the operation.

When this time arrives, I always introduce into the root of the tooth, as nearly to the apex as I cam get it, a small pledget of cotton, well balled up, and saturated with the oil of creosote or eucalyptus, and proceed to fill the root with either Hill's stopping or gutta-percha.

If either of these preparations are used as thoroughly as they are capable of being, you can make the whole thing moisture proof, from end to end, so to speak.

But after all, doing the very best we can, diagnosing and prognosing such cases, let no one be so sanguine as to think that perfect success will crown our efforts on all occasions. Indeed, do the very best we can, it is no uncommon occurrence for there to be an attack of periostitis, more or less severe, upon closing the apertures of the tooth.

For fear of this, it is always better to give the case the benefit of a doubt, and not finish the operation at once, but let it rest on trial for a few days, or even weeks.

If you have a pulpless tooth with an abscess, the first thing to be done is to cure the abscess (if you can). For the sake of convenience we will suppose it entirely amenable to treatment. Having accomplished this, the operation to finish the work is precisely the same as the first tooth of which I have spoken.

It needs no argument to prove that a tooth with a live pulp in it is infinitely to be preferred to one that has not. But when the circumstances are such that we cannot have our way in these matters, the next best thing is in order. I believe that a large number of such teeth as we have been considering, with due care, may be rendered serviceable for a number of years, and not a few destined for a long life.

### "Treatment of Pulpless Teeth."

BY A. G. ROSE, D.D.S.

Read Before Mississippi Valley Society of Dental Surgeons.

*Mr. President and Gentlemen:* —

This paper is presented to you in a very brief form, and is intended to introduce the topic for discussion. We find pulpless teeth in four different conditions. The first case is one where there has been little or no pain attending the death of the pulp, the surrounding parts in a normal condition, tooth being somewhat discolored. These cases are usually discovered by the dentist, much to the surprise of the patient, who has experienced no discomfort, and is astonished at the condition the tooth presents. It is needless for me to go into detail as to the methods of procedure here, each of us, no doubt, having some favorite plan valuable to us from long experience and habit in using our particular disinfectant. It is in the thorough use of the disinfectant or germicide, that good results can be obtained in treatment of this condition. Let me here call your attention to some of the new and old remedies used for the purpose: Sanitas oil, Eugenol oil, peroxide of hydrogen, bichloride of mercury, carbolic acid, and iodine equal parts, salicylic acid, iodoform, creosote, carbonate of soda, tannin. In this condition is it best practice to push through the apical foramen and draw blood, carrying not only the point of broach through, but also particles of debris with it, which, no doubt, will create a slight inflammation if nothing more, not to speak of wounding the pericementum and leave a weak point for pericemental trouble to originate in the future? It seems better practice to give these cases rest and gentle treatment, complete removal of all disintegrated pulp tissue, and disinfect to the utmost. Should pulp tissue not be removed soon after the devitalization, or before putrescence occurs salphureted and phosphoreted hydrogen gas arising from the decaying pulp, may escape through the foramen, particularly when the cavity of decay is closed, and irritation and inflammation of the tissues surrounding and adjacent to the end of the root may be induced. This irritation may be incited, not only by the gas, but by particles of the pulp, or any foreign matter which may be forced through the foramen during the cleansing of pulp chambers. Following these causes, and often arising during treatment, are we confronted with this second condition: "Pulpless Teeth, accompanied by Perecementitis." The opening up of the pulp cavity or the removal of dressings, if treatment for disinfection is in progress, will generally give relief in a few hours. Depletion of the gum, the careful and complete removal of all calcareous deposits about the necks of the teeth and necrosed portion of alveolar border will sometimes be all that is necessary. The applications to the gum over the affected tooth of a mixture of equal parts aconite and iodine tincture, painting the face along the course of nerves and blood vessels with a mixture of chloroform and tinct. of aconite, using capsicum plaster, and continuing them for some time after relief is obtained, will greatly assist in effecting a cure. In this and the two following conditions, constitutional treatment will be of great benefit, and is oftentimes necessary to produce any beneficial results. Often in cases of pericementitis, treatment directed to the system will be of greater avail than local applications. Anodynes, tonics, and cathartics, are used with a view to the effect desired. In all stages of treatment, we cannot do better than to insist upon the continued use of mouth washes, the list of which is headed to-day by Phenol Sodique and Listerine. When pericementitis continues, the result is usually the formation of a sac at the end of the root which is a product of plastic pericementitis as seen under the microscope. If the sac is composed of dense fibrous connective tissue, the inner surface of the sac is not smooth, but largely provided with irregular protrusions or papillary outgrowths of a myxomatous structure crowded with inflammatory elements. This is the third condition, Pulpless Teeth with an Abcess, without an external opening. In this stage where the pulp has been dead for some time and abscess in its incipiency, or, where it has gone to such an extent as to prevent rebuilding of the tissues surrounding the end of the root, it is sometimes necessary to carry a small drill just through the foramen, so as to make a fresh wound and secure healing by first intention. So long as putrescent tissue remains in the pulp chamber, and the mephitic gas arising from it escapes through the apical foramen, so long may the production of pus corpuscles continue. This transformation of the tissue that is destroyed into "pus" should be changed and the parts gotten into such condition as to favor a return to normal action, else the abscess may become chronic. There are two methods in vogue for the treatment of this stage. Injecting the disinfectant through the root and into the saccule by means of a finely pointed syringe, and carrying a piece of floss silk or cotton saturated with the remedy, into the canal and through the foramen with a broach. The cloride of zinc can be used to advantage in this latter way, and solutions of carbolic acid, bichloride of mercury, peroxide of hydrogen, nitrate of silver, in the former. When the application is made by means of placing pieces of silk or cotton in root canal, and the medicinal effect of the remedy desired for a few days, it is best to seal the cavity of decay with a preparation of gutta percha and wax, or some good temporary stopping instead of the old method of a pellet of cotton saturated with gum sandarac. You receive not only the blessings of your patient, but achieve better results in treatment. In the last condition of "Pulpless Teeth with an Abscess, and a Fistulous Opening," it is proper after removing all disintegrated pulp tissue, enlarging the pulp canal and fistulous opening, to force an escharotic or disinfectant through the apical foramen into the sac and the fistulous opening if possible, this will bring about a healthy condition of the tissues involved at the point of the root; will prevent further disintegration, the continuace of "pus" production, and the rebuilding of the "gum" tissue at the external opening. In bad cases of chronic abscess, it will be necessary to enlarge the fistulous opening, and with a burr or abscess knife cut away all necrosed alveolus saccule and portions of tooth substance, if necrosed. This is treatment which rarely fails to effect a complete rebuilding of new tissue. Care must be taken that the opening upon the "gum" does not close before reformation of tissue has taken place. The use of Dr. Farrar's syringe in this condition is well known to you all. An invention by Dr. Watt, called the Exhaust Syringe, is particularly adapted to treat cases of this kind. By its means medicine in proper quantity can be drawn directly through the pulp cavity, sac and sinus, and is what is termed an artificial leech. This is a great improvement on the old method of injecting medicine through root, sac, and sinus, and the consequent excoriation of the soft tissues of the mouth, which is entirely prevented by the exhaust syringe. The exhaustive action of the artificial leech is designed to promote the collapse of the sac walls in contradistinction to the distending effects of the common force pump process, and may presumably hasten the obliteration of the sac.

